# Construction and validation of a prognostic model with RNA binding protein-related mRNAs for the HBV-related hepatocellular carcinoma patients

**DOI:** 10.3389/fonc.2022.970613

**Published:** 2022-09-23

**Authors:** Shaohua Xu, Hui Liu, Renyun Tian, Jiahui Xie, Su Chen, Junyun Luo, Haizhen Zhu, Yirong Wang, Zhaoyong Li

**Affiliations:** ^1^ Hunan Provincial Key Laboratory of Medical Virology, Institute of Pathogen Biology and Immunology of College of Biology, Hunan University, Changsha, China; ^2^ Research Center of Cancer Prevention & Treatment, Translational Medicine Research Center of Liver Cancer, Hunan Cancer Hospital, Changsha, China; ^3^ Bioinformatics Center, College of Biology, Hunan University, Changsha, China; ^4^ Research Institute of Hunan University in Chongqing, Chongqing, China

**Keywords:** hepatocellular carcinoma, hepatitis B virus, RNA binding protein, mRNA, prognostic model

## Abstract

Hepatocellular carcinoma (HCC) is a common malignancy worldwide with poor clinical outcomes, and the infection of hepatitis B virus (HBV) is the leading cause of this disease. Mounting evidence shows that RNA binding proteins (RBPs) can modulate the progression of cancers. However, the functions and clinical implications of RBP-related mRNAs in HBV-related HCC remain largely unclear. Therefore, we aim to develop a prognostic model based on the RBP-related mRNAs for HBV-related HCC patients. Firstly, we identified 626 differentially expressed RBP-related mRNAs in the HBV-related HCC through the Pearson correlation analysis. Subsequently, the Kaplan-Meier survival, univariate, Least Absolute Shrinkage and Selection Operator (LASSO), and multivariate Cox regression analyses were used to construct a prognostic model comprised of five RBP-related mRNAs. Furthermore, the patients were categorized into the high- and low-risk groups by the prognostic model and the patients in the high-risk group had a poor prognosis. Additionally, the prognostic model was an independent predictor of prognosis, and the accuracy of the prognostic model was proved by the receiver operator characteristic (ROC) analysis. Furthermore, the functional enrichment analysis revealed that various cancer-promoting processes were enriched in the high-risk group. Taken together, our study may provide the HBV-related HCC biomarkers of prognosis to improve the clinical outcomes of patients.

## Introduction

Hepatocellular carcinoma (HCC) comprises approximately 75%-85% of all cases of primary liver cancer, which is the sixth most frequently diagnosed cancer and the third leading cause of cancer-related death worldwide in 2020, with an estimated 906,000 new cases and 830,000 deaths (1). However, chronic infection of the Hepatitis B virus (HBV) remains the leading cause of liver cirrhosis and hepatocellular carcinomas (2–4). Moreover, HBV reactivation is common following surgical resection, transarterial chemoembolization (TACE), radiotherapy, local ablation therapy, and systemic therapy for HCC, as well as in patients who receive no specific HCC treatment (5). Although the treatment methods and techniques have been constantly improved, the patients with HBV-related HCC still suffer from a poor postoperative prognosis (6–8) and a high frequency of recurrence or metastasis (6, 9–12). Furthermore, how genetic alterations drive cancer phenotypes and relapse in HBV-related HCC remains largely unknown. Therefore, accurate risk stratification for HBV-associated HCC is still necessary.

RNA binding proteins (RBPs) are evolutionarily conserved proteins that can bind their RNA targets through their functional RNA-binding domains (13). Consequently, a series of mRNA metabolic processes were regulated, including pre-mRNA splicing, capping, polyadenylation, RNA modification, transportation, localization, translation, and degradation (14). Aberrant expression and function of RBPs, which are usually observed in various cancers, result in the promotion or suppression of carcinogenesis, development, and recurrence (15). For example, *ENO1* is highly expressed in human HCC tissues and promotes liver cancer progression by functioning as an RNA-binding protein and recruiting CNOT6 to promote the degradation of *IRP1* mRNA, which leads to down-regulated expression of Mfrn1 and suppression of mitochondrial iron-induced ferroptosis (16).

Intriguingly, an increasing number of studies have demonstrated that RBPs are involved in transcription control by binding to chromatin. Some chromatin regulators, the proteins that can directly regulate chromatin functions and transcription, have been identified as RBPs (17). WDR43, an RBP essential for embryonic pluripotency, is recruited by promoter-associated noncoding and/or nascent RNA to open chromatin and promotes Pol II pause release and transcription elongation at thousands of its target genes (18). QKI5, another RBP, has also been found to bind DNA and activate transcription of some monocytic differentiation-associated genes (19). Besides, the RBP HNRNPL can stabilize the binding of pol II to hemidesmosome and extracellular matrix genes and promote their transcription, therefore contributing to epidermal renewal (20). Recently, a large-scale RBP ChIP-seq analysis by Xiao, *et al.* revealed that 30 RBPs exhibit extensive and specific interactions with chromatin in liver cancer cells HepG2 (21). Moreover, all of these chromatin-associated RBPs show a universal preference for active gene promoters and may directly participate in transcriptional control *via* interaction with transcription factors (21–23). Through their interaction with active chromatin, RBPs collectively play a significant role in regulating gene expression.

Although RNA binding protein-related prognostic signatures for HCC (24–26) or HBV-related HCC (27), as well as an immune-related RNA-binding protein signature for liver cancer (28), have been developed for the prognosis evaluation of patients, the RBPs-related mRNA predictive risk model for HBV-related HCC remains unknown. Therefore, our study aimed to construct a reliable prognostic signature based on mRNAs related to 30 RBPs, which are generally localized on active chromatin regions and participate in transcription regulation (21). We have systematically identified mRNAs that were correlated with the 30 chromatin-associated RBPs in HBV-related HCC. With five RBP-related mRNAs, we established a reliable prognostic risk model, which can accurately predict the prognosis of HBV-related HCC patients. Additionally, the functional enrichment analysis revealed the risk model was tightly connected with some cancer-promoting pathways, such as proliferation-related pathways, hypoxia, epithelial-mesenchymal transition, and angiogenesis pathways. In sum, our study developed an RBP-related mRNA signature that may provide a theoretical reference for the prognostic estimation or clinical treatment of HBV-related HCC patients.

## Materials and methods

### Data acquisition and pre-processing

We downloaded the gene expression data normalized by upper quartile fragments per kilobase per million reads (FPKM-UQ) and clinical information of HBV-related HCC patients (159 paired normal and tumor tissues) from the NODE website (https://www.biosino.org/node/, accessed on 12 August 2021) (29). The proteomic data of the above samples were collected from the Clinical Proteomic Tumor Analysis Consortium (CPATC) database (https://cptac-data-portal.georgetown.edu/study-summary/S049, accessed on 14 September 2021). The 159 patients from the NODE website were used as a training cohort. Additionally, we also downloaded the gene expression data (FPKM-UQ normalized RNA-seq) and clinical information of HCC patients (50 normal and 371 tumor tissues) from the TCGA database (https://portal.gdc.cancer.gov/, accessed on 4 September 2021). Furthermore, according to the clinical information of liver cancer patients of the TCGA database downloaded in the UCSC Xena database (http://xena.ucsc.edu/, accessed on 16 September 2021), 145 HBV-positive patients with HCC were screened out as the test cohort, including 60 patients with HBV only and 85 patients with HBV and HCV (hepatitis C virus). The gene expression level was further transformed to log_2_ (FPKM-UQ+1). Besides, we also downloaded the gene expression microarray data of GSE14520 from the Gene Expression Omnibus (GEO) database (https://www.ncbi.nlm.nih.gov/geo, accessed on 16 March 2022). The 221 HBV-positive patients with complete survival information were used as the validation cohort.

### Identification of RBP-related mRNAs in HBV-related HCC

We used the R package limma (v3.44.3) (30) to analyze the differential expression of genes between normal and HBV-related HCC tissues, and the genes with |log_2_(fold change)| > 1.5 and *p*.adjust< 0.001 were considered differentially expressed. Then, we screened out mRNAs that were significantly up-regulated or down-regulated in the RNA-seq data from the NODE and TCGA databases. Subsequently, the Pearson method was used to analyze the correlation between the 30 chromatin-related RBPs in HepG2 cells (21) and the differentially expressed mRNAs, and the mRNAs with |R| > 0.3 and *p*< 0.05 were identified as RBP-related mRNAs.

### Establishment of a prognostic model composed of RBP-related mRNAs in the training cohort

The patients with overall survival (OS)< 30 days were excluded to ensure the accuracy of the analysis. Therefore, we obtained a training cohort of 158 HBV-related HCC patients from the NODE database and a test cohort containing 145 HBV-related HCC patients from the TCGA database. According to the optimal cut-point of the RBP-related mRNA expression determined by the “surv_cutpoint” function in the survminer package (survminer package version v.0.4.8, https://rpkgs.datanovia.com/survminer/index.html, accessed on 6 November 2020), the training cohort was divided into the high- and low-expression groups. The Kaplan-Meier survival analysis and log-rank test were used to assess the difference in OS between the high- and low-expression groups using the survival package (31). Subsequently, we selected the RBP-related mRNAs that were up-regulated in tumors and were associated with poor prognosis of patients or down-regulated in tumors and were related to a good prognosis.

Next, the survival package was used to perform the univariate Cox regression analysis to obtain OS-related mRNAs (*p<* 0.001). The LASSO Cox regression analysis was utilized to filter some mRNAs through the R package glmnet (32) to prevent the model from overfitting. Multivariate cox regression was used to construct the prognostic model and the risk score of each patient was calculated according to the formula: risk score = $\Sigma_{i=1}^{n}Coefi*Expi$ . The “Coef” indicates the coefficient of each mRNA derived from the multivariate Cox analysis and “Exp” represents the expression level of each mRNA.

### Evaluation and validation for the prognostic value of the RBP-related mRNA prognostic model in the training and test cohorts

According to the optimal cut-point of the risk score, the training and test cohorts were divided into a high-risk group and a low-risk group, respectively. The Kaplan-Meier survival analysis and log-rank test were used to assess the difference in OS between the high- and low-risk groups. The R package survivalROC (survivalROC package version v.1.0.3, https://cran.r-project.org/web/packages/survivalROC/index.html, accessed on 22 December 2020) was employed to draw the time-dependent ROC curves for the risk score to evaluate the prediction accuracy of the prognostic model. Then we drew the scatterplots of risk scores and survival status of patients between high- and low-risk groups. The expression heatmaps of the mRNAs in the model were drawn by the R package pheatmap (https://cran.r-project.org/web/packages/pheatmap/index.html/). The RBP-mRNA co-expression network was constructed to explore the relationships between the RBPs and their related mRNAs. The Cytoscape software (33) (v.3.8.2, https://cytoscape.org/, accessed on 22 October 2020) was performed to visualize the co-expression network.

### The relationship between risk score and clinical characteristics

We compared the difference in risk scores *via* the Wilcoxon test between the patients with early-stage (TNM stage I-II) and late-stage (TNM stage III-IV), as well as the difference in risk scores between the alive and dead patients. Moreover, we used the Kaplan-Meier survival analysis to evaluate the differences in OS between the high- and low-risk groups in different subgroups classified by clinical characteristics, including TNM stage (stage I-II, stage III-IV), viral infection (HBV, both HBV and HCV). The univariate Cox and multivariate Cox analyses were used to assess the independent prognostic value of the risk score and clinical characteristics (age, gender, and TNM stage). The ROC curve analysis was conducted to compare the accuracy in predicting the prognosis of risk score and other clinical features.

### Functional enrichment analysis

Functional annotations of differentially expressed genes (DEGs) between the high- and low-risk groups were performed by the R package ClusterProfiler (34). The terms of Gene Ontology (GO) with *p*.adjust< 0.05 were considered to be significantly enriched. The gene set enrichment analysis (GSEA) between the high- and low-risk groups was conducted based on the hallmark gene sets (h.all.v7.4.symbols.gmt) through the GSEA software (v.4.1.0; http://www.broadinstitute.org/gsea/index.jsp, accessed on 18 March 2021). The hallmark pathways with the normalized enrichment score |NES | > 1 and FDR< 0.05 were considered to be significantly enriched.

### Verification for the prognostic value of the RBP-related mRNA prognostic model in the validation cohort

According to the optimal cut-point of the risk score, the validation cohort was divided into a high-risk group and a low-risk group. The Kaplan-Meier survival analysis and log-rank test were used to assess the difference in OS between the high- and low-risk groups. The time-dependent ROC curves for the risk score were drawn to evaluate the prediction accuracy of the prognostic model. We compared the difference in risk scores *via* the Wilcoxon test and Kruskal-Wallis test between the patients with different TNM stages, as well as the difference in risk scores between the alive and dead patients. The Cox analyses were used to assess the independent prognostic value of the risk score and clinical characteristics. R package GSVA (35) was performed based on the hallmark gene sets to calculate the enrichment score for each pathway for each patient. Limma package was used to search hallmark pathways with significantly different enrichment scores between the high- and low-risk groups (*p*< 0.05).

### Cell culture

The human HCC cell lines HepG2, Hep3B, and PLC were grown in Dulbecco’s modified Eagle’s medium (DMEM) supplemented with 10% fetal bovine serum (FBS). HepAD38 cell line stably transformed with two copies of HBV genome was maintained in MEM medium supplemented with 10% FBS, 0.3 mg/ml tetracycline, and 400 mg/ml G418 (GIBCO BRL/Life Technologies) (36). The HLCZ01 cell line was cultured with DMEM/F12 medium supplemented with 10% FBS, 40 ng/mL of dexamethasone, and 10 ng/mL of EGF in collagen-coated tissue culture plates as described previously (37).

### RNA preparation and qRT-PCR determination for mRNAs

A total of 23 HCC samples and adjacent normal tissues were collected from HCC patients who underwent surgical resection from June 2019 to November 2021 in the Hunan Cancer Hospital. All patients involved provided written informed consent. This research was approved by the Ethics Committee of Hunan Cancer Hospital.

For the assessment of RBP-related mRNAs’ expression in tissue, total RNA was extracted from the tissue and cells samples using TRIzol reagent (Thermo Fisher Scientific). Reverse transcription of total RNA was performed using PrimeScript™ RT reagent Kit with gDNA Eraser (Takara, Kusatsu, Japan) to avoid genomic DNA contamination. Quantitative PCR was conducted using SYBR^®^ Premix Ex Taq™ II (Tli RNaseH Plus) (Takara) with cDNA product, and the primers for PCR are listed in **Supplementary Table 1**. RBP-related mRNA expressions were analyzed using the 2^-ΔΔCT^ method. GAPDH mRNA was used as a control. Outliers were removed for the accuracy of the results, and the T-test (after F-test) was utilized to analyze the differences in mRNA expression levels between the HCC tissues and adjacent tissues.

For hepatic HBV DNA detection, total DNA was isolated from liver tissue samples of HCC patients and real-time PCR for HBV DNA was performed as described previously (38). The primers used for PCR detection to HBV DNA were 5’-CACCTCTGCCTAATCATC-3’ (sense) and 5’-GGAAAGAAGTCAGAAGGCAA-3’ (antisense). GAPDH was used as the internal control. The primers used for PCR detection to GAPDH DNA were 5’- GCACCGTCAAGGCTGAGAAC-3’ (sense) and 5’-TGGTGAAGACGCCAGTGGA-3’ (antisense). The relative fold change of HBV DNA was calculated using the 2^-ΔCT^ method.

### ELISA

HBs antigens in culture supernatants were measured using commercial ELISA kits (Shanghai Kehua Bio-Engineering Company, KHB) according to the manufacturer’s manuals.

### Plasmids construction, lentivirus production, and cell counting assay

The shRNAs targeting human F11 and PSRC1 were designed and subcloned into the lentiviral vector pLKO.1-puro (Sigma-Aldrich) (**Supplementary Table 2**). The lentiviral plasmids were co-transfected with packaging plasmids pMD2.G and psPAX2 into HEK293T cells for 24h or 48h. Lentivirus expressing shRNAs or pLKO.1-puro (non-target control, NTC) were collected and added into the culture medium of Hep3B and PLC cells in the presence of polybrene. Cell numbers were determined by trypan blue counting and data were presented as mean ± SD.

### Western blotting

Cell lysates were prepared in RIPA buffer [10% SDS, 20% NP-40, 0.5M EDTA, 5M NaCl, and 1.25M Tris-Cl (pH 8.0)] supplemented with proteasome inhibitors. Equal amounts of protein were separated on SDS-PAGE gels and then transferred to PVDF membranes (Amersham, GE, USA). Membranes were incubated with specific primary antibodies: anti-F11 (45895-1; Signalway Antibody); anti-PSRC1 (32879-1; Signalway Antibody). Anti-β-actin (66009-1-Ig; Proteintech) served as the loading control. HRP-conjugated anti-mouse or anti-rabbit secondary antibodies were used, and signals were detected using Western ECL substrate (Beyotime).

### Statistical analysis

Data were analyzed on R software (version v.4.0.2, https://www.r-project.org/, accessed on 22 June 2020). The limma package was used to identify the DEGs between the two groups. The Pearson method was used to analyze the correlation between RBPs and differentially expressed mRNAs. The differences in risk scores between different groups were calculated with the Wilcoxon test or Kruskal-Wallis test. Unless otherwise stated, *p*-value<0.05 were considered significant.

## Results

### Identification of differentially expressed mRNAs associated with RBPs in HBV-related HCC

**Figure 1:**
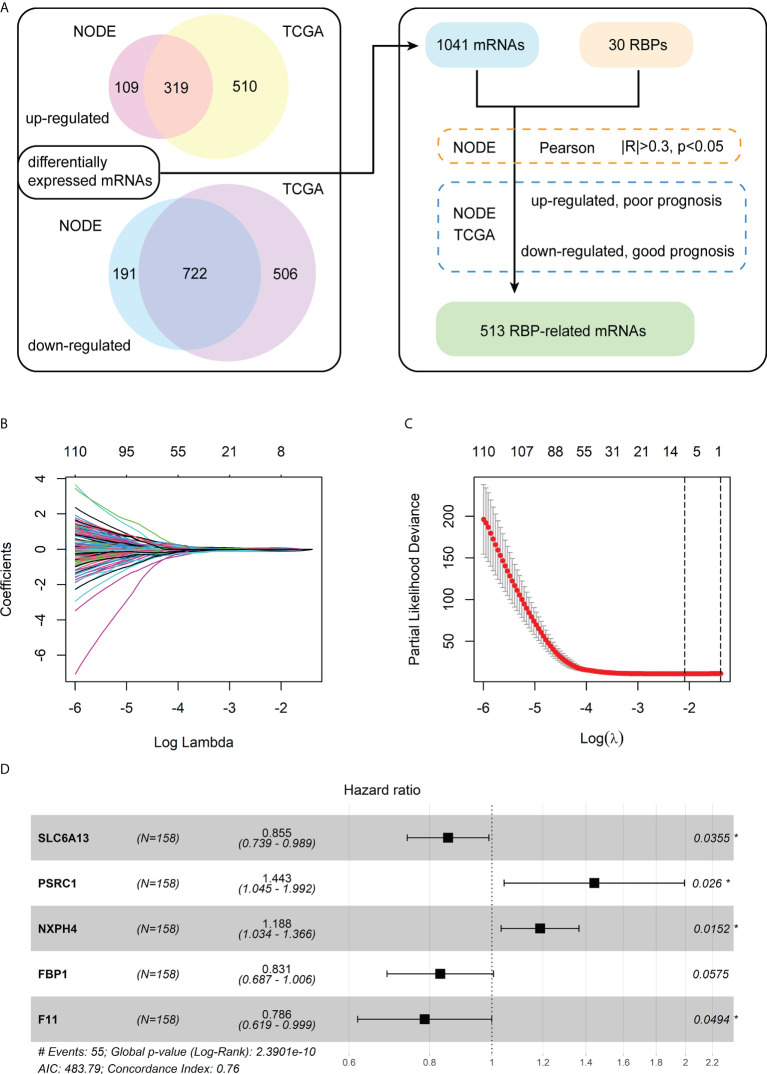
Identification of the differentially expressed mRNAs associated with RBPs and construction of a prognostic model. **(A)** The flowchart for identification of RBP-related mRNAs. **(B)** The LASSO coefficient profiles of the RBP-related mRNAs related to OS. **(C)** The cross-validation plot of LASSO regression Cox analysis shows the optimal parameter selection with minimum criteria (the lambda value corresponding to the black dashed line on the left). **(D)** Forest plot of the five RBP-related mRNAs obtained *via* the multivariate Cox regression analysis.

The analysis process of differentially expressed mRNAs related to RBPs is shown in the flowchart (**Figure 1A**). First, we identified 1,341 differentially expressed mRNAs between the 159 paired normal and HBV-related HCC from the NODE database. Meanwhile, we detected 2,057 differentially expressed mRNAs between the 50 normal and 145 HBV-related HCC from the TCGA database. Then, we chose 1,041 differentially expressed mRNAs that were both significantly up-regulated and down-regulated in the tumor tissues of the NODE and TCGA databases. Subsequently, a total of 626 mRNAs were identified as RBP-related mRNAs by expression correlation analysis of the 30 chromatin-related RBPs and 1,041 differentially expressed mRNAs (|R| > 0.3, *p<* 0.05).

Furthermore, for the 626 RBP-related mRNAs, we screened out mRNAs whose expression levels are negatively correlated with the prognosis of patients, according to the Kaplan-Meier survival analysis in both the training cohort (NODE) and test cohort (TCGA). Then, 513 shared mRNAs were identified between the training and test cohorts, with 207 upregulated and 306 downregulated.

### Construction of a prognostic model consisting of five RBP-related mRNAs in the training cohort

Among 513 RBP-related mRNAs, 135 mRNAs were further screened to be associated with OS through univariate Cox analysis in the training cohort (*p<* 0.001). Then, the LASSO Cox analysis was performed to avoid the overfitting of the model. As shown in **Figures 1B, C**, the upper abscissa represents the number of mRNAs with non-zero coefficients under the corresponding lambda. As the lambda value increased, there were fewer mRNAs with non-zero coefficients (**Figure 1B**). As a result, 11 mRNAs were obtained according to the optimal parameter (the lambda value corresponding to the dotted line on the left in the figure) selection with minimum criteria in the LASSO model (**Figure 1C**). Subsequently, the multivariate Cox analysis was performed to build a prognostic model consisting of five RBP-related mRNAs (**Figure 1D**). Next, we calculated the risk score of each patient based on the coefficients (**Table 1**) and the expression of these five mRNAs.

**Table 1:** The coefficients of 5 RBP-related mRNAs by the multivariable Cox regression analysis in the training cohort.

Gene symbol	Ensembl ID	Genomic location	Coefficient
F11	ENSG00000088926	Chr4: 186,266,189-186,289,681	-0.240334189
FBP1	ENSG00000165140	Chr9: 94,603,133-94,640,249	-0.184718481
SLC6A13	ENSG00000010379	Chr12: 220,621-262,873	-0.156826316
NXPH4	ENSG00000182379	Chr12: 57,216,794-57,226,449	0.172572929
PSRC1	ENSG00000134222	Chr1: 109,279,556-109,283,186	0.366513978

The reference genome version used for the Genomic location was GRCh38. Chr, chromosome.

The forest plot of the univariate Cox regression analysis for the five RBP-related mRNAs in the training cohort showed three mRNAs (F11, FBP1, and SLC6A13) were protective factors for the prognosis of HBV-HCC patients (HR< 1), whereas two mRNAs (NXPH4 and PSCR1) were risk factors (HR > 1) (**Supplementary Figure 1A**). The Pearson correlation analysis (|R| > 0.3, p< 0.05) showed that 20 of 30 chromatin-related RBPs were tightly associated with the five mRNAs in the prognostic model. We also constructed an RBP-mRNA co-expression network to visualize their relationships (**Supplementary Figure 1B**). The detailed correlations of these mRNAs with RBPs and risk types are shown in the Sankey plot (**Supplementary Figure 1C**).

### The expression of the five mRNAs of the prognostic model and survival analysis

The boxplot indicated the mRNAs expression of F11, FBP1, and SLC6A13 were significantly down-regulated in the HBV-related HCC tissues compared to normal tissues from the NODE database, whereas the NXPH4 and PSRC1 were significantly up-regulated (**Supplementary Figure 2A**). Similarly, the expression levels of these 5 mRNAs were dysregulated between the normal and HBV-related HCC tissues from the TCGA database (**Supplementary Figure 3A**). The survival analyses based on expression levels of these 5 mRNAs were conducted to further clarify their prognostic role in the HBV-related HCC. The mRNAs F11, FBP1, and SLC6A13 were associated with the good prognosis of the HBV-related HCC patients in the training cohort (**Supplementary Figures 2B–D**) and test cohort (**Supplementary Figures 3B–D**). In contrast, the mRNAs NXPH4 and PSRC1 were associated with the poor prognosis of the patients in the training cohort (**Supplementary Figures 2E, F**) and test cohort (**Supplementary Figures 3E, F**).

### Assessment and validation for the prognostic value of the RBP-related mRNA model

**Figure 2:**
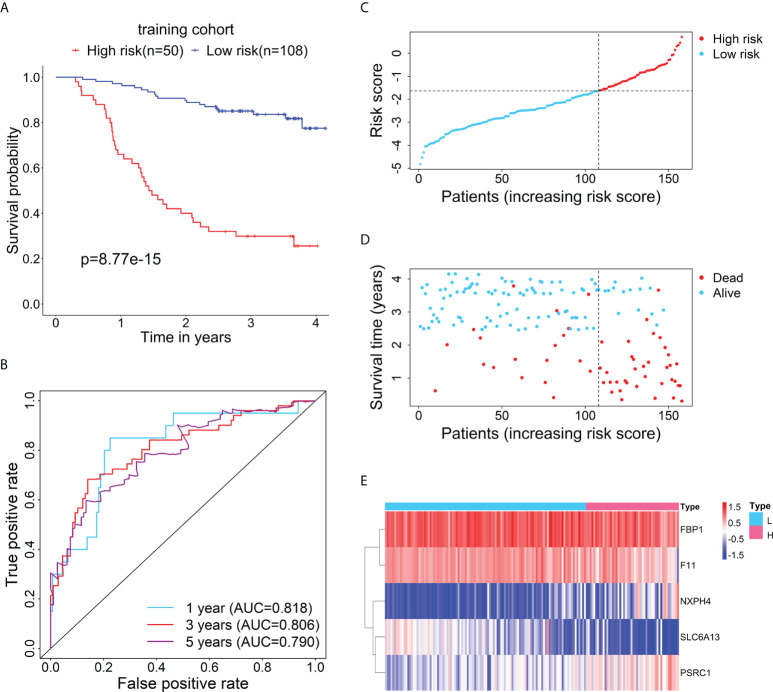
Evaluation of the prognostic performance of the RBP-related risk model in the training cohort. **(A)** Kaplan–Meier curve shows the difference in OS between the high- and low-risk groups in the training cohort. **(B)** The 1-, 3-, and 5-year ROC curves based on the risk score show the accuracy of the prognostic prediction of the RBP-related risk model. **(C)** The distribution of risk scores of patients in the high- and low-risk groups. **(D)** The distribution of survival time and status of patients in the high- and low-risk groups. **(E)** Heatmap of clustering analysis for the expression of five RBP-related mRNAs in the high- and low-risk groups.

Firstly, the training cohort was divided into the high-risk (n = 50) and low-risk (n = 108) groups based on the optimal cut-point of risk score (risk score = -1.6378). Kaplan-Meier survival curve demonstrated that the survival rate of the high-risk group was significantly lower than that of the low-risk group (**Figure 2A**). Additionally, time-dependent ROC curves were drawn to verify the accuracy of the prognostic model, and 1-, 3- and 5-year AUC values reached 0.818, 0.806, and 0.790, respectively (**Figure 2B**). The distributions of risk score and survival status between the high- and low-risk groups are plotted in **Figures 2C, D**. The heatmap showed the expression levels of the five RBP-related mRNAs between the high- and low-risk groups (**Figure 2E**).

**Figure 3:**
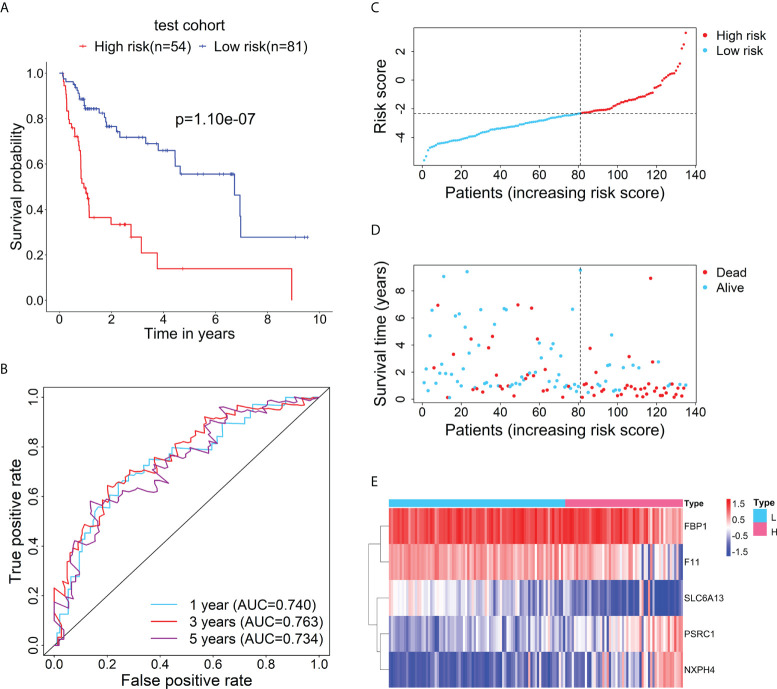
Verification for the prognostic performance of the RBP-related risk model in the test cohort. **(A)** Kaplan–Meier curve shows the difference in OS between the high- and low-risk groups in the test cohort. **(B)** The 1-, 3-, and 5-year ROC curves based on the risk score show the accuracy of the prognostic prediction of the RBP-related risk model. **(C)** The distribution of risk scores in the high- and low-risk groups. **(D)** The distribution of survival time and status of patients in the high- and low-risk groups. **(E)** Heatmap of clustering analysis for the expression of five RBP-related mRNAs in the high- and low-risk groups.

Furthermore, to test the prognostic performance of the RBP-related mRNA model, the test cohort was categorized into the high-risk (n = 54) and low-risk (n = 81) groups according to the optimal cut-point of risk score (risk score = -2.3237). Consistent with the previous result, Kaplan-Meier survival curve demonstrated that the patients in the high-risk group had shorter OS than that of the low-risk group (**Figure 3A**). ROC curves showed the 1-, 3- and 5-year AUC values were 0.740, 0.763, and 0.734, respectively (**Figure 3B**). The distributions of risk score and survival status between the high- and low-risk groups are plotted in **Figures 3C, D**. The heatmap showed the expression levels of the five RBP-related mRNAs between the high- and low-risk groups (**Figure 3E**).

### Correlation of the risk score of the prognostic model with clinical characteristics

**Figure 4:**
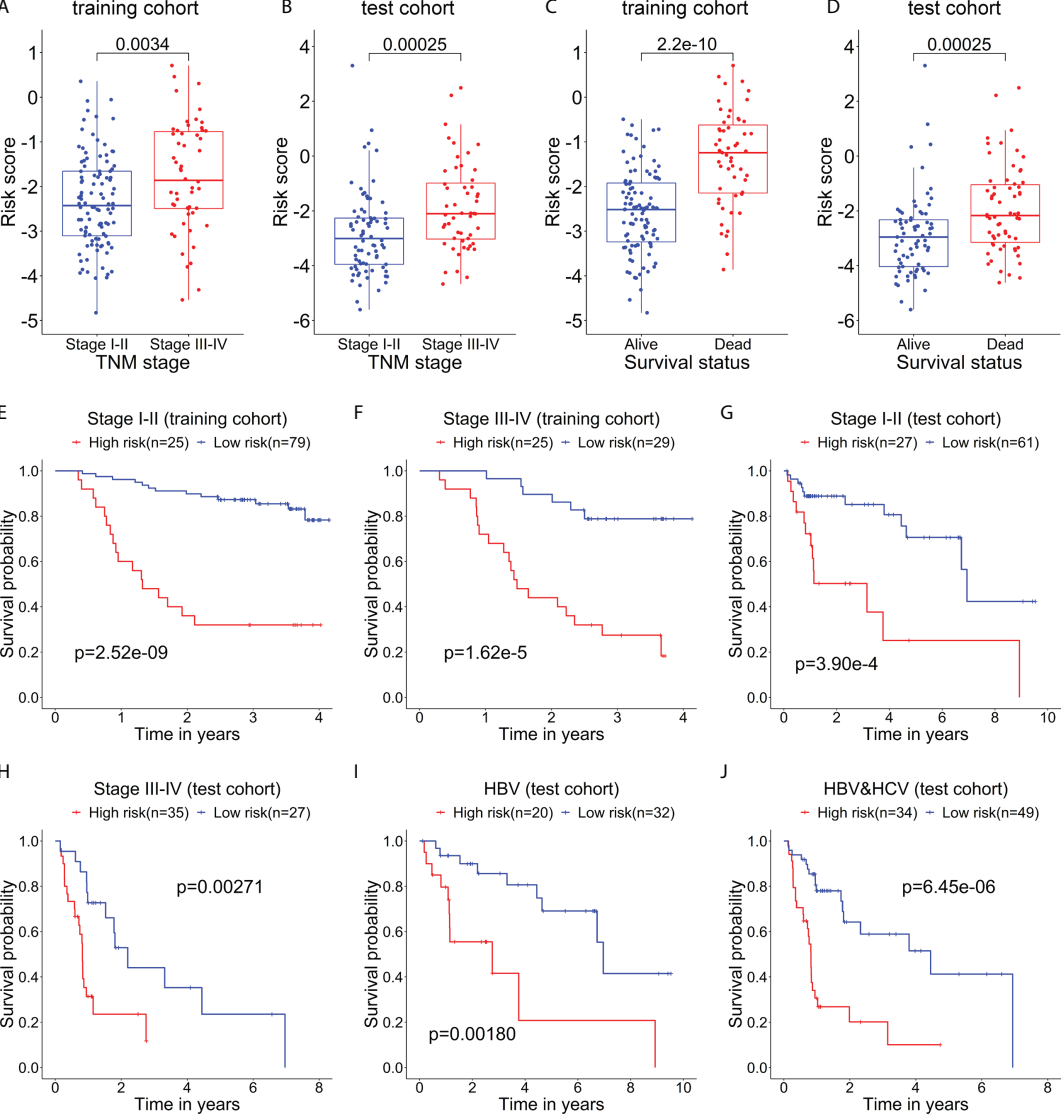
The relationship between the risk score and clinical characteristics of patients. The box plots show that the risk scores of patients with stage III-IV are significantly higher than those of patients with stage I-II in the training cohort **(A)** and test cohort **(B)**. The box plots show that the risk scores of dead patients are significantly higher than those of alive patients in the training cohort **(C)** and test cohort **(D)**. Kaplan–Meier curves show the difference in OS between the high- and low-risk groups in two subgroups of the training cohort, including Stage I-II **(E)** and Stage III-IV **(F)**. Kaplan–Meier curves show the difference in OS between the high- and low-risk groups in several subgroups of the test cohort, including Stage I-II **(G)**, Stage III-IV **(H)**, HBV (only HBV-positive) **(I)**, and HBV-HCV (both HBV- and HCV-positive) **(J)**.

The relationship between the risk score of the 5-mRNA model and clinical characteristics was evaluated to explore whether the model was associated with the clinical characteristics. The patients with late-stage (TNM stage III-IV) had higher risk scores than patients with early-stage (TNM stage I-II) in the training cohort (**Figure 4A**) and test cohort (**Figure 4B**). The dead patients also had higher risk scores than alive patients in the training cohort (**Figure 4C**) and test cohort (**Figure 4D**).

Then, the stratification analysis was used to evaluate differences in OS between the high- and low-risk groups of various subgroups stratified by the clinical characteristics. In the training cohort, the prognosis of patients with high-risk scores was worse than that of patients with low-risk scores in the subgroups classified by the early and later stages (**Figures 4E, F**). Additionally, in the test cohort, the patients in the high-risk group had a poorer prognosis than that of patients in the low-risk group in the subgroups categorized by the clinical characteristics, including TNM stage I-II, TNM stage II-IV, HBV-positive, and HBV- and HCV-positive (**Figures 4G–J**).

### The prognostic model served as an independent prognostic factor for HBV-related HCC patients

**Figure 5:**
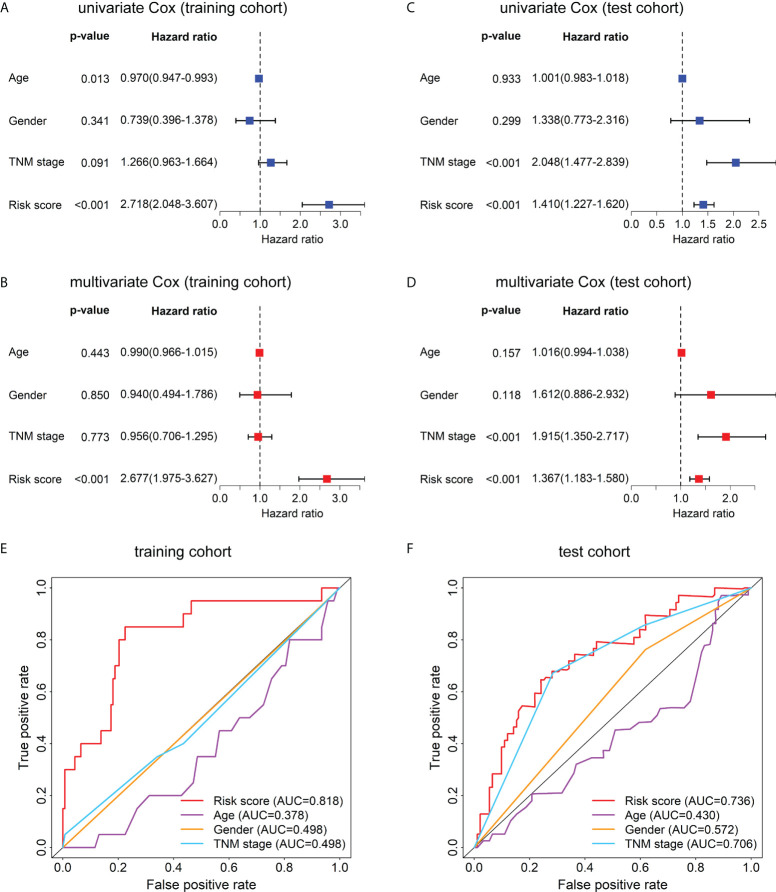
Univariate Cox, multivariate Cox, and ROC curve analyses of the risk score and clinical characteristics in the training and test cohorts. Forest plots of the univariate **(A)** and multivariate **(B)** Cox regression analyses of risk score and clinical characteristics in the training cohort. Forest plots of the univariate **(C)** and multivariate **(D)** Cox regression analyses of the risk score and clinical characteristics in the test cohort. **(E)** The ROC curves at 1-year OS of the risk score and clinical characteristics in the training cohort. **(F)** The ROC curves at 1-year OS of the risk score and clinical characteristics in the test cohort.

To evaluate whether this model can be used as an independent prognostic factor for HBV-related HCC patients, we performed univariate Cox analysis and multivariate Cox analysis. First, in the training data set, the result of the univariate factor Cox analysis showed that the risk score of the model was highly correlated with the patients’ prognosis (**Figure 5A**). The result of the multivariate Cox analysis indicated the risk score can be used as an independent prognostic factor for the patients (**Figure 5B**). Similarly, the univariate (**Figure 5C**) and multivariate Cox analysis (**Figure 5D**) in the test cohort also demonstrated the risk score can act as an independent prognostic factor. Moreover, in the training cohort (**Figure 5E**) and test cohort (**Figure 5F**), the AUC values of the risk score were also higher than those of other clinical characteristics such as age, gender, and TNM stage.

### Functional enrichment analysis

**Figure 6:**
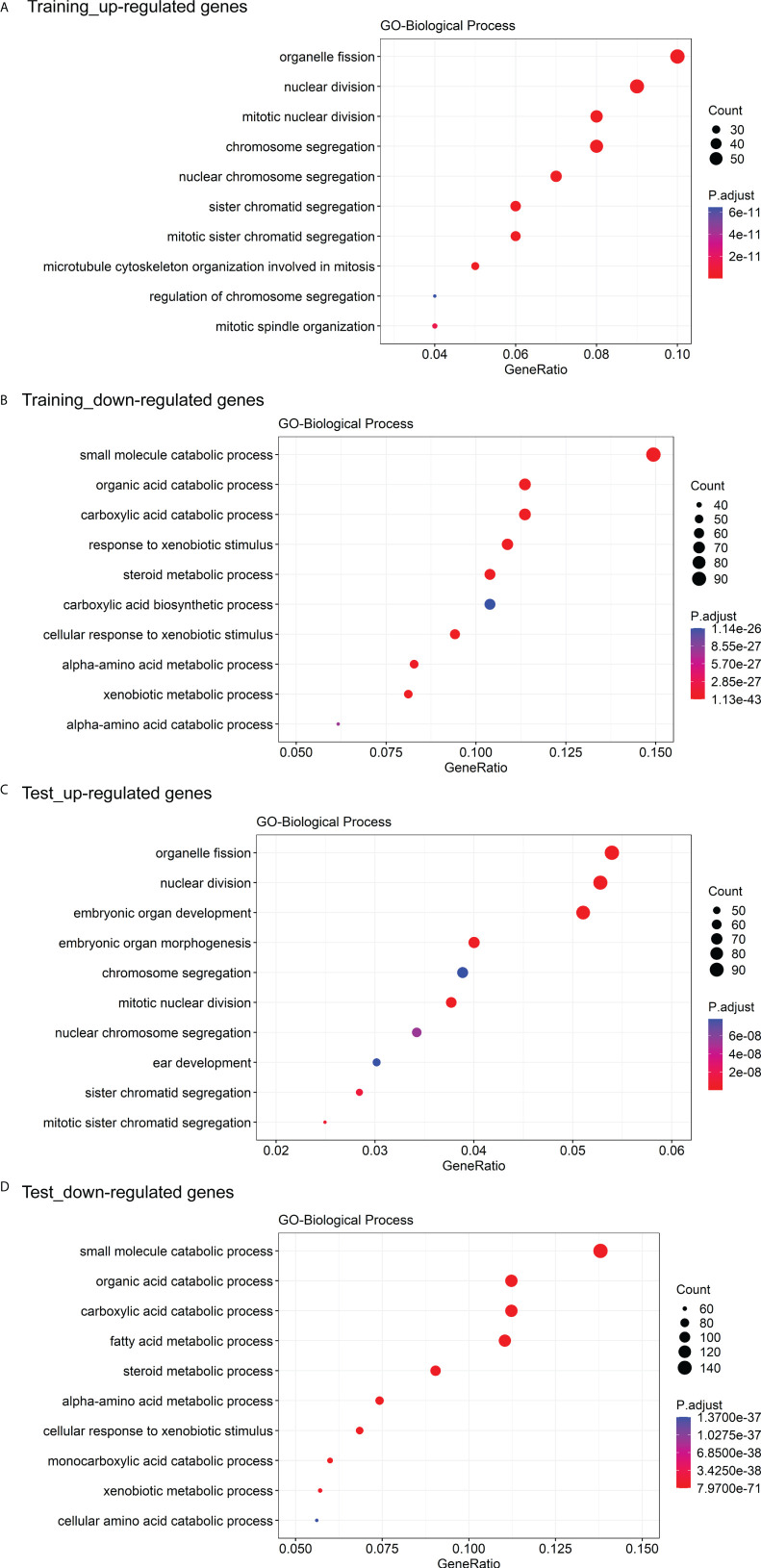
GO enrichment analysis of DEGs between the high- and low-risk groups. **(A)** The top 10 GO biological processes with the significant enrichment of the up-regulated genes between the high- and low-risk groups in the training cohort. **(B)** The top 10 GO biological processes with the significant enrichment of the down-regulated genes between the high- and low-risk groups in the training cohort. **(C)** The top 10 GO biological processes with the significant enrichment of the down-regulated genes between the high- and low-risk groups in the test cohort. **(D)** The top 10 GO biological processes with the significant enrichment of the down-regulated genes between the high- and low-risk groups in the test cohort.

**Figure 7:**
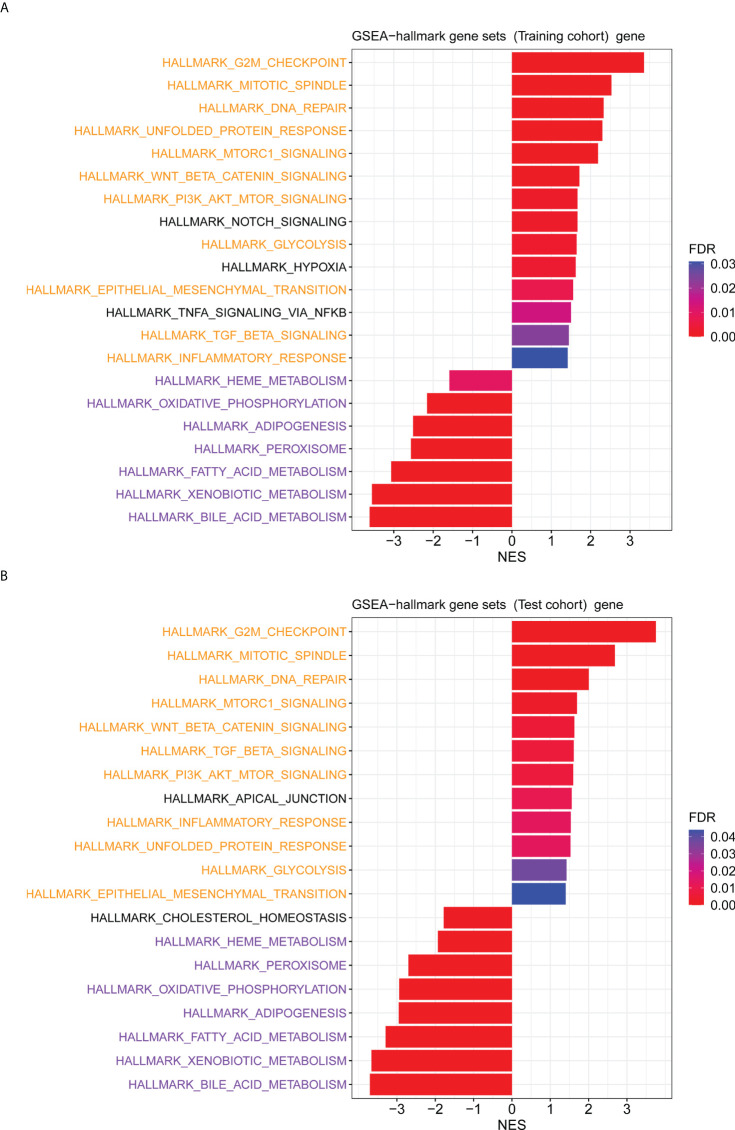
The GSEA analysis for genes between the high- and low-risk groups. **(A)** The hallmark pathways that were significantly enriched in the high- and low-risk groups based on the transcriptome data in the training cohort. **(B)** The hallmark pathways were significantly enriched in the high- and low-risk groups based on the transcriptome data in the test cohort. Pathways marked in yellow are some pathways that were both enriched in the high-risk group of training and test cohorts, and pathways marked in purple are some pathways that were both enriched in the low-risk group of training and test cohorts.

The GO enrichment and GSEA analyses were employed to further explore the potential biological prognostic model involved in HBV-related HCC patients. The up-regulated DEGs between the high- and low-risk groups were mainly enriched in cell cycle-related pathways (**Figures 6A, C**). However, down-regulated DEGs were mainly enriched in some metabolic-related pathways (**Figures 6B, D**). The GSEA analyses for genes in the training and test cohorts suggested that some pathways closely related to cancer were enriched in the high-risk group, such as “HALLMARK G2M CHECKPOINT”, “HALLMARK MITOTIC SPINDLE”, “HALLMARK DNA REPAIR”, “HALLMARK EPITHELIAL MESENCHYMAL TRANSITION”, “HALLMARK GLYCOLYSIS”, and “HALLMARK INFLAMMATORY RESPONSE” pathways (**Figures 7A, B**). Conversely, “HALLMARK OXIDATIVE PHOSPHORYLATION” and some other metabolic pathways were enriched in the low-risk group (**Figures 7A, B**). Similarly, the GSEA analysis for proteins in the test cohort revealed that the high-risk group was enriched in the pathways associated with cancer (**Supplementary Figure 4A**), whereas the low-risk group was enriched in the pathways relevant to metabolism (**Supplementary Figure 4B**).

### Verification for the prognostic value of the RBP-related mRNA model

**Figure 8:**
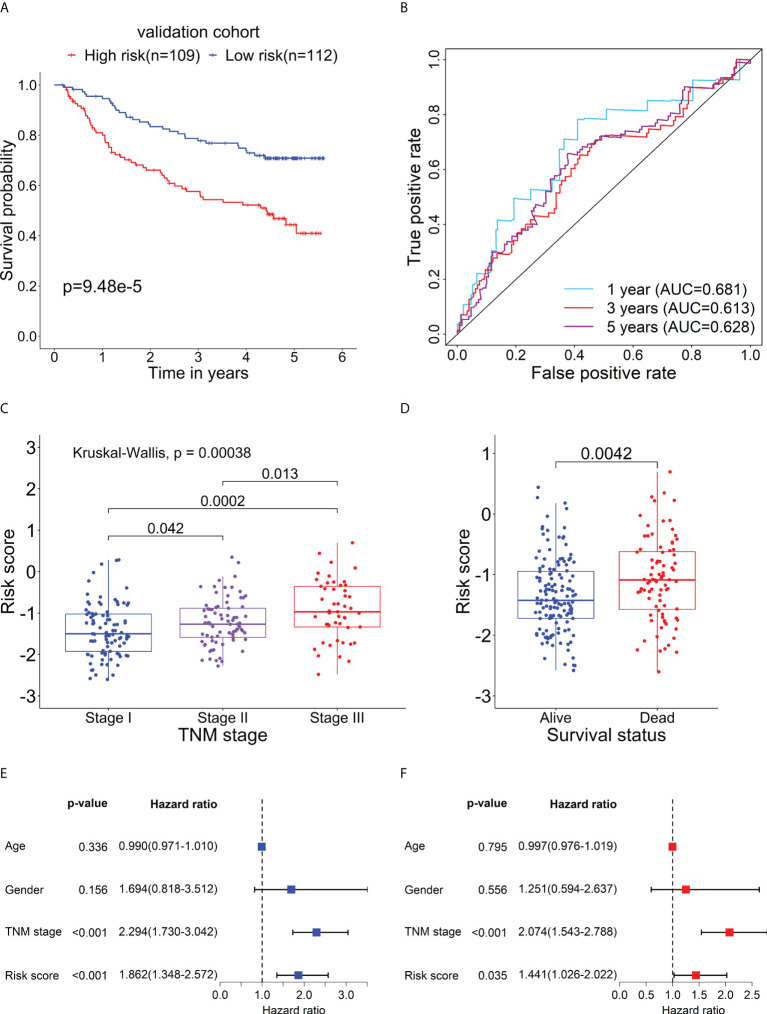
Verification for the prognostic performance of the RBP-related risk model in the validation cohort. **(A)** Kaplan–Meier curve shows the difference in OS between the high- and low-risk groups in the validation cohort. **(B)** The 1-, 3-, and 5-year ROC curves based on the risk score show the accuracy of the prognostic prediction of the RBP-related risk model. **(C)** The box plot shows the difference in risk scores of patients with different TNM stages. **(D)** The box plot shows the difference in risk scores of patients with different survival statuses. Forest plots of the univariate **(E)** and multivariate **(F)** Cox regression analyses of risk score and clinical characteristics in the validation cohort.

To further evaluate the prognostic performance of the RBP-related mRNA model, we divided the validation cohort into the high-risk (n = 109) and low-risk (n = 112) groups based on the optimal cut-point of risk score (risk score = -1.2607). Similar to previous results, the Kaplan-Meier survival curve indicated that the patients in the high-risk group had a lower survival rate than that in the low-risk group (**Figure 8A**). ROC curves of risk score showed the 1-, 3- and 5-year AUC values were 0.681, 0.612, and 0.627, respectively (**Figure 8B**). As shown in **Figure 8C**, as the tumor stage increased, the risk score also increased significantly. The dead patients also had higher risk scores than alive patients in the validation cohort (**Figure 8D**). The univariate (**Figure 8E**) and multivariate Cox analysis (**Figure 8F**) supported that the risk score could serve as an independent prognostic factor. The result of Gene Set Variation Analysis (GSVA) demonstrated that the metabolic-related pathways were enriched in the low-risk group and the proliferation-related pathways were enriched in the high-risk group (**Supplementary Figure 5**).

### Determination of the five RBP-related mRNAs’ expression levels in HCC

**Figure 9:**
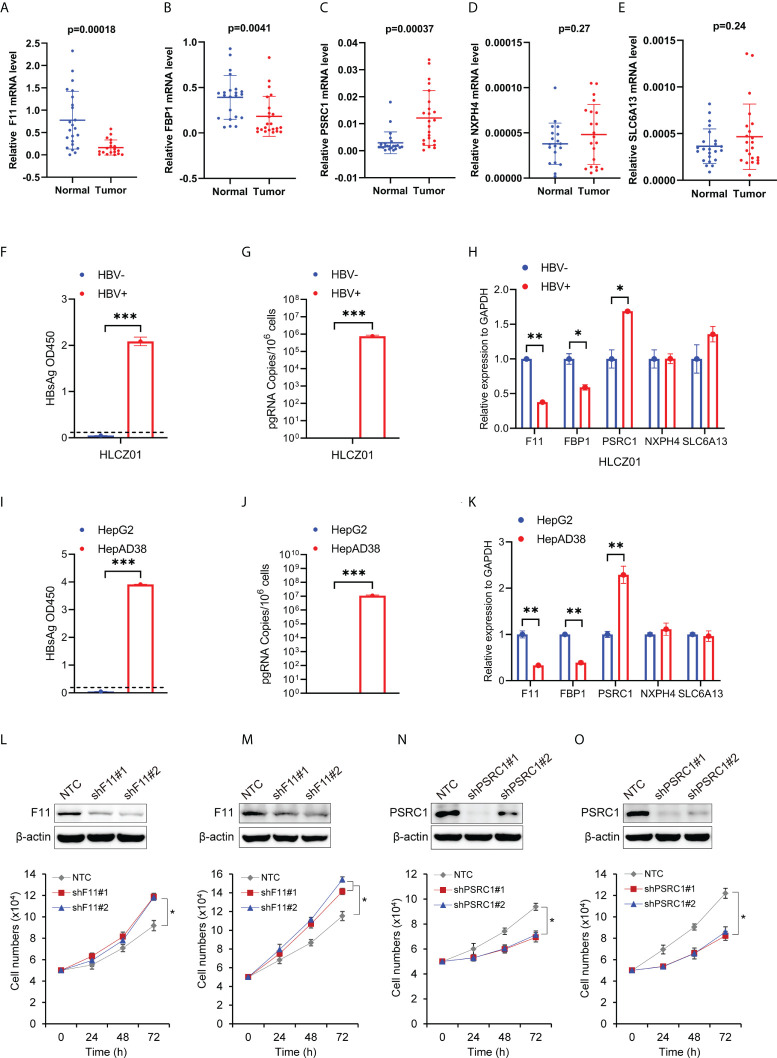
The expression levels of the five RBP-related mRNAs in HCC tissues and cells, and their effect on cell proliferation. The scatter plots show F11 **(A)**, FBP1 **(B)**, PSRC1 **(C)**, NXPH4 **(D)**, and SLC6A13 **(E)** mRNA expression levels in HCC tissues (Tumor) and adjacent normal tissues (Normal). **(F)** HBsAg in the supernatant of HBV-infected (HBV+) or uninfected (HBV-) HLCZ01 cells was detected by ELISA. **(G)** The viral pregenomic RNA level determined by real-time PCR was shown as the number of HBV pregenomic RNA copies per 10^6^ cells. **(H)** The mRNA abundance of F11, FBP1, SLC6A13, NXPH4, and PSRC1 was assayed in HBV+ or HBV- HLCZ01 cells. The differences of HBsAg, the viral pregenomic RNA level, and the five RBP-related mRNAs’ expression levels were also compared between HepG2 (HBV-) and HepAD38 (HBV+) cell lines **(I–K)**. The expression levels of the five RBP-related mRNAs were normalized by GAPDH mRNA. Cell growth was determined by trypan blue counting in Hep3B **(L)** or PLC **(M)** cells expressing NTC or shF11 (shF11#1 or shF11#2). Knockdown efficiency of F11 was verified by Western blotting. Cell growth was also determined by trypan blue counting in Hep3B **(N)** or PLC **(O)** cells expressing NTC or shPSRC1 (shPSRC1#1 or shPSRC1#2). Knockdown efficiency of PSRC1 was verified by Western blotting. *P<0.05 between the indicated groups; **P<0.01 between the indicated groups; ***P<0.001 between the indicated groups.

To examine the expression of the five RBP-related mRNAs in HCC by experiment, we performed the quantitative real-time PCR with RNAs extracted from 23 pairs of HCC tissues and adjacent tissues in which the presence of HBV DNAs was confirmed among the genomic DNAs (**Supplementary Figure 6**). Our experimental results indicated that, compared to adjacent tissues, F11 and FBP1 were significantly down-regulated (**Figures 9A, B**), whereas PSRC1 was significantly up-regulated (**Figure 9C**) in HCC tissues. However, in our experiment, we didn’t observe the expression change of NXPH4 and SLC6A13 mRNA levels between HCC tissues and adjacent tissues (**Figures 9D, E**), both of whom were lower than that of the other three mRNAs.

### The expression levels of the five RBP-related mRNAs in HBV- and HBV+ Hepatoma cells

To further examine the effect of HBV on the five RBP-related mRNAs, we compared their expression in HBV- and HBV+ HCC cells, including HBV- and HBV+ HLCZ01 cells, as well as HepG2 and HepAD38 cells (**Figures 9F, G, I, J**). It was found that the expression levels of F11 and FBP1 mRNAs were significantly decreased, while the expression level of PSRC1 mRNA was significantly increased in the presence of HBV (**Figures 9H, K**). However, NXPH4 and SLC6A13 mRNA levels were not significantly different between HBV- and HBV+ HCC cells.

### The effect of RBP-related mRNAs on cell proliferation of HBV-related HCC cell lines

To explore the function of RBP-related mRNAs in HBV-related HCC, we knocked down the expression of F11 and PSRC1 with shRNAs in Hep3B and PLC, two cell lines that contain Hepatitis B viral DNA and secrete HBsAg. Our experimental results show that cell proliferation increased after the knockdown of F11 (**Figures 9L, M**). However, PSRC1 knockdown leads to decreased cell proliferation (**Figures 9N, O**). Therefore, F11 and PSRC1 may play a suppressive and enhancing effect on cell proliferation of HBV-containing HCC cell lines, respectively.

## Discussion

HBV is a hepatotropic virus that can cause persistent infection and chronic hepatitis, which may ultimately lead to cirrhosis and HCC (39, 40). Despite the HBV vaccination and antiviral therapies are effective, HBV-related HCC remains the current major factor for liver cancer and mortality (4, 41). Therefore, the search for reliable prognostic biomarkers to improve clinical outcomes for patients is urgent. Multiple RBPs have been demonstrated to be related to the prognosis of HCC patients (24, 25, 42), including HBV-related HCC (27). Although emerging evidence implicates gene expression has been regulated by RBP at the transcriptional level (19, 21–23), the prognostic values of genes associated with chromatin-relevant RBPs in HBV-related HCC remain largely unknown. Thence, we herein aimed to develop a prognostic model for HBV-positive HCC patients based on the mRNAs related to chromatin-relevant RBPs.

In this study, we firstly screened out 1,041 mRNAs that were differentially expressed between normal and HBV-related HCC tissues. Subsequently, we identified 626 differentially expressed mRNAs related to the 30 RBPs associated with chromatin based on the proteomic and RNA-seq data. Furthermore, 135 RBP-related mRNAs were confirmed to be associated with the prognosis of HBV-related HCC patients through the survival and univariate Cox regression analyses in the training cohort. Afterward, combining the LASSO and multivariate Cox regression analysis, a panel of five RBP-related mRNAs was selected to construct a prognostic model in the training cohort. The risk score of each patient in the training, test, and validation cohorts were calculated based on the expression levels of the five genes. Moreover, we evaluated and verified the prognostic value of this model in the training, test, and validation cohorts. The patients were divided into the high- and low-risk subgroups based on the optimal cut-point of risk score, patients in the high-risk group had a worse prognosis. Multivariate Cox regression analysis indicated that the risk score was an independent risk factor for OS. ROC curve analysis suggested that the prognostic model has high accuracy in the prognostic prediction for HBV-related HCC patients.

GSEA analyses between the high- and low-risk groups indicated that some cancer-related pathways were enriched in the high-risk group, including the proliferation- and metastasis-related pathways, while some metabolic pathways were enriched in the low-risk group (**Figure 7** and **Supplementary Figures 4–5**). Previous studies have shown that HCC can be broadly categorized into the proliferation and nonproliferation subgroups, and the proliferation subgroup that tends to have a more aggressive phenotype was related to a poor prognosis of HCC patients (43, 44). Thus, in our study, we considered the high-risk group as the proliferation subgroup and the low-risk group as the metabolism subgroup (nonproliferation subgroup). Consistent with previous findings, the HBV-related HCC patients of the proliferation subgroup had a worse clinical outcome than those of the metabolism subgroup (1). Concretely, the hypoxia, glycolysis, epithelial-mesenchymal transition, and angiogenesis pathways were enriched in the high-risk group, but the oxidative phosphorylation pathway was enriched in the low-risk group (**Figure 7** and **Supplementary Figures 4–5**). Metabolic reprogramming is an important biological process by which cancer can acquire the material and energy, and one hallmark of it is the increase in aerobic glycolysis with the decrease in oxidative phosphorylation (45). The hypoxic environment promotes anaerobic glycolysis in cancer and the cancer cells prefer the glycolytic anaerobic metabolism even under normal circumstances (46). Additionally, some studies suggested that the hypoxia-inducible factor proteins could promote angiogenesis in HCC tumors by inducing the expression of proangiogenic factors (47). As the tumor cells in the high-risk group tend to and angiogenesis phenotypes, the antiangiogenics might be suitable for the treatment of patients in the high-risk group (48, 49). To sum up, we explored the possible reasons for the different outcomes of patients in the high- and low-risk groups and potential therapy for patients in the high-risk group.

Our study revealed that the five mRNAs that constituted the prognostic model were all significantly dysregulated and related to the OS of HBV-related HCC patients. Among them, F11, FBP1, and SLC6A13 were down-regulated in HBV-related HCC and can act as the protective factors for the prognosis of HBV-related HCC patients. In contrast, the NXPH4 and PSRC1 were down-regulated in HBV-related HCC and can serve as the prognostic risk factors. The previous study revealed that the mRNA and protein levels of FBP1 were significantly decreased in HCC tissues relative to normal tissues (50). Mounting evidence also shows that FBP1 can inhibit the occurrence and development of HCC through the metabolic pathways (51, 52). Furthermore, the low FBP1 expression can predict a poor clinical outcome in HCC (51, 53). Recent research found that NXPH4 promotes the proliferation and migration of lung cancer cells (54). The bioinformatics-based study uncovered that the mRNA expression level of NXPH4 was significantly up-regulated in HCC (55). The high mRNA levels of PSRC1 are associated with poor survival of HCC patients (56) and the oncoprotein DDA3 encoded by the PSRC1 gene is downregulated by p53 (57). Experimentally, we validated significant down-regulation of F11 and FBP1, but up-regulation of PSRC1 mRNA level in HCC tissues. However, we were unable to detect the expression variation of NXPH4 and SLC6A13 mRNA in HCC, possibly as a result of their low expression levels in both HCC tissues and adjacent tissues. Interestingly, we found long-term survival of HBV leads to down-regulated expression levels of F11 and FBP1 mRNAs but up-regulated PSRC1 mRNA in HBV-replicating hepatocellular carcinoma cell lines (**Figures 9F–K**). Moreover, F11 decreases but PSRC1 increases cell proliferation of HBV-containing HCC cells (**Figures 9L–O**), indicating their potential tumor-suppressive and promoting function in HCC development, respectively. Collectively, the above evidence suggests that the five mRNAs may play pivotal roles in the occurrence and progression of HCC.

To sum up, based on the 5 RBP-related mRNAs, our study developed and validated the prognostic model for HBV-related HCC patients. Furthermore, in clinical applications, measuring the expression levels of only five mRNAs can provide an accurate prognosis for patients and is beneficial to reduce costs. Although our study used three independent cohorts, there are still some limitations. Firstly, a relatively small number of HBV-positive patients were used in this study. Thus, further validation in the other cohort containing a relatively large number of patients can verify the accuracy of the model. Secondly, future studies about the molecular mechanisms of the five mRNAs in the model will further support our study. Nonetheless, the prognostic model may serve as the biomarker for HBV-related HCC patients.

## Conclusions

In conclusion, this study identified 626 differentially expressed mRNAs associated with 30 chromatin-related RBPs in the HBV-related HCC. A 5-mRNA panel derived from these mRNAs was used to establish the prognostic model for HBV-related HCC patients. Additionally, the accuracy of the prognostic models was validated separately in the independent datasets. Therefore, the models may be useful for clinical prognostic risk stratification.

## Data availability statement

The original contributions presented in the study are included in the article/**Supplementary Material**. Further inquiries can be directed to the corresponding authors.

## Ethics statement

This research was approved by the Ethics Committee of Hunan Cancer Hospital. All patients involved provided written informed consent.

## Author contributions

SX, HL, RT, JX, SC, and JL performed the analysis and experiment. SX, HL, and RT wrote the manuscript. ZL, YW, and HZ revised and completed the final manuscript. All authors have read and agreed to the published version of the manuscript.

## Funding

This work was financially supported by the Natural Science Foundation of Chongqing (cstc2021jcyj-msxmX0401), the Natural Science Foundation of Hunan (2022JJ40048, 2022JJ30182), the Natural Science Foundation of Changsha (kq2202153), the National Natural Science Foundation of China (81772552), and the Fundamental Research Funds for the Central Universities of China (531118010140).

## Conflict of interest

The authors declare that the research was conducted in the absence of any commercial or financial relationships that could be construed as a potential conflict of interest.

## Publisher’s note

All claims expressed in this article are solely those of the authors and do not necessarily represent those of their affiliated organizations, or those of the publisher, the editors and the reviewers. Any product that may be evaluated in this article, or claim that may be made by its manufacturer, is not guaranteed or endorsed by the publisher.
