# Supplementary material for: Construction and validation of a prognostic model with RNA binding protein-related mRNAs for the HBV-related hepatocellular carcinoma patients
Source: Front Oncol. 2022 Sep 23;12:970613. doi: 10.3389/fonc.2022.970613 (PMC9539435; doi:10.3389/fonc.2022.970613)
Supplement: Supplementary file 1 [file DataSheet_1.docx]

**›Supplementary Materials**

| **Supplementary Table 1. Primers for qRT-PCR detection of mRNAs** | | | |
| --- | --- | --- | --- |
| Genes | Primers | Sequences (5'→3') |  |
| F11 | Forward | GAATGGCCCAAAGAATCTCA |  |
|  | Reverse | CTGTGCCTGCAGCTTTGTAG |  |
| FBP1 | Forward | CTCTGCACAGCAGTCAAAGC |  |
|  | Reverse | TAACCAGGTCGTTGGAGAGG |  |
| SLC6A13 | Forward | CTGGGGAGATCATTGGCTTA |  |
|  | Reverse | ACTGGCCTAGTGCTGTCTCC |  |
| NXPH4 | Forward | CTCAGCCGCCAGAGAAGAT |  |
|  | Reverse | TTCCGGACTCTGGTATCTGG |  |
| PSRC1 | Forward | CGGGCTACATCTGGAAAGAG |  |
|  | Reverse | TCGATCGGGTAAGAGGAGAA |  |
| GAPDH | Forward | GAAGGTGAAGGTCGGAGTCA |  |
|  | Reverse | AATGAAGGGGTCATTGATGG |  |
| pgRNA | Forward | CTCAATCTCGGGAATCTCAATGT |  |
|  | Reverse | TGGATAAAACCTAGCAGGCATAAT |  |

| **Supplementary Table 2. Oligonucleotide sequences of shRNAs** | | |
| --- | --- | --- |
| Genes | Primers | Sequences (5'→3') |
| shF11#1 | Forward | CCGGCCACCCAAGATGTTTACTCTTCTCG  AGAAGAGTAAACATCTTGGGTGGTTTTTG |
|  | Reverse | AATTCAAAAACCACCCAAGATGTTTACTC  TTCTCGAGAAGAGTAAACATCTTGGGTGG |
| shF11#2 | Forward | CCGGCTAGACATGAAGGGCATAAACCTC  GAGGTTTATGCCCTTCATGTCTAGTTTTTG |
|  | Reverse | AATTCAAAAACTAGACATGAAGGGCATA  AACCTCGAGGTTTATGCCCTTCATGTCTAG |
| shPSRC1#1 | Forward | CCGGTCCTGTTTCCCAGCGACTAAACTCG  AGTTTAGTCGCTGGGAAACAGGATTTTTG |
|  | Reverse | AATTCAAAAATCCTGTTTCCCAGCGACTA  AACTCGAGTTTAGTCGCTGGGAAACAGGA |
| shPSRC1#2 | Forward | CCGGTCCCAGGACCTACCAGGTAAACTCG  AGTTTACCTGGTAGGTCCTGGGATTTTTG |
|  | Reverse | AATTCAAAAATCCCAGGACCTACCAGGT  AAACTCGAGTTTACCTGGTAGGTCCTGGGA |

**
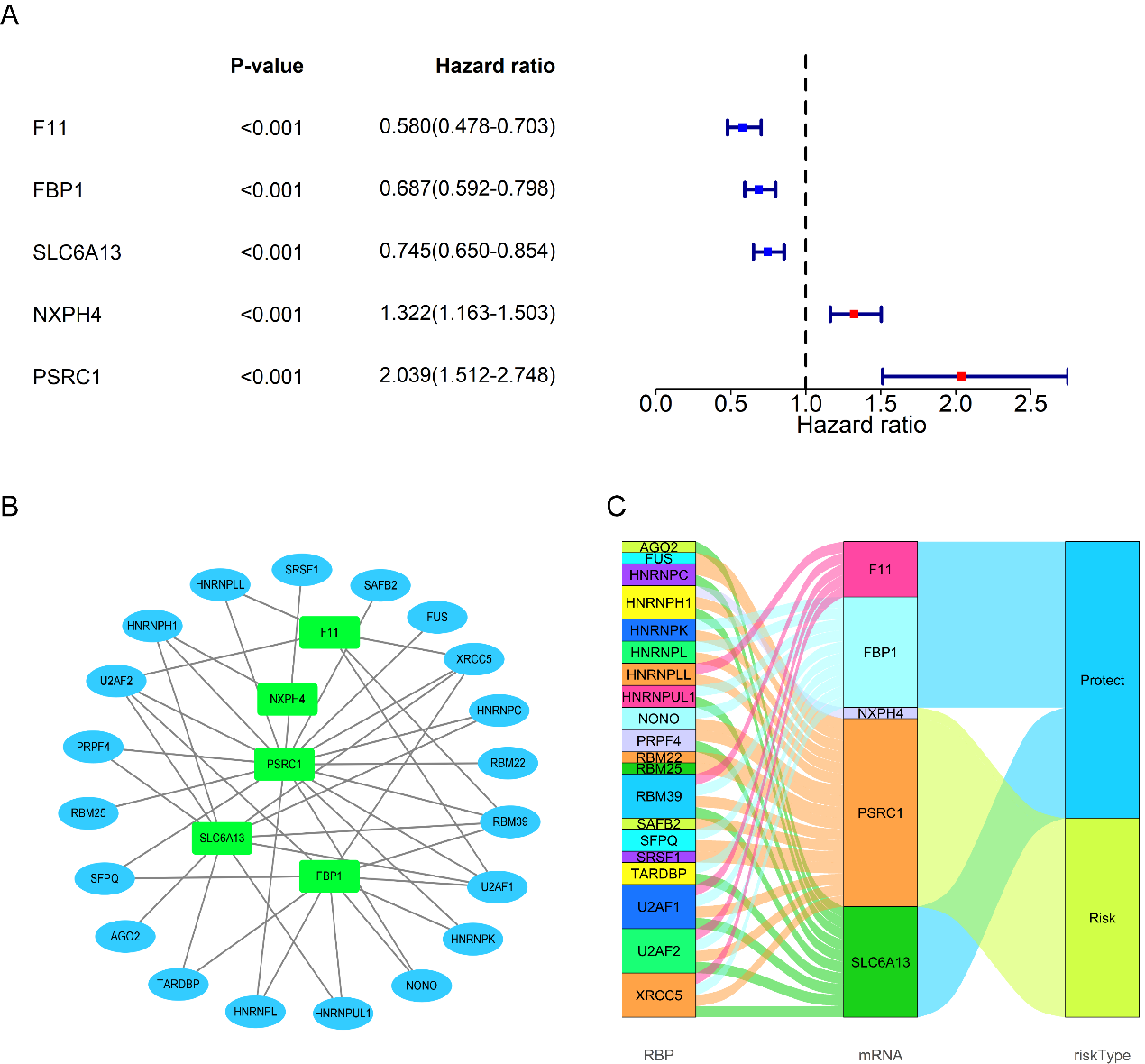
**

**Supplementary Figure 1. The relationship of five mRNAs in the prognostic model and their related 20 RBPs in the training cohort**

**(A)** Forest plot of the univariate Cox regression analysis of five RBP-related mRNAs that constructed the prognostic model. **(B)** The co-expression network comprised five mRNAs in the prognostic model and their related 20 RBPs. Green nodes represent the mRNAs and blue nodes represent the RBPs. **(C)** The Sankey diagram shows the relationships of the five mRNAs with 20 RBPs and risk types.


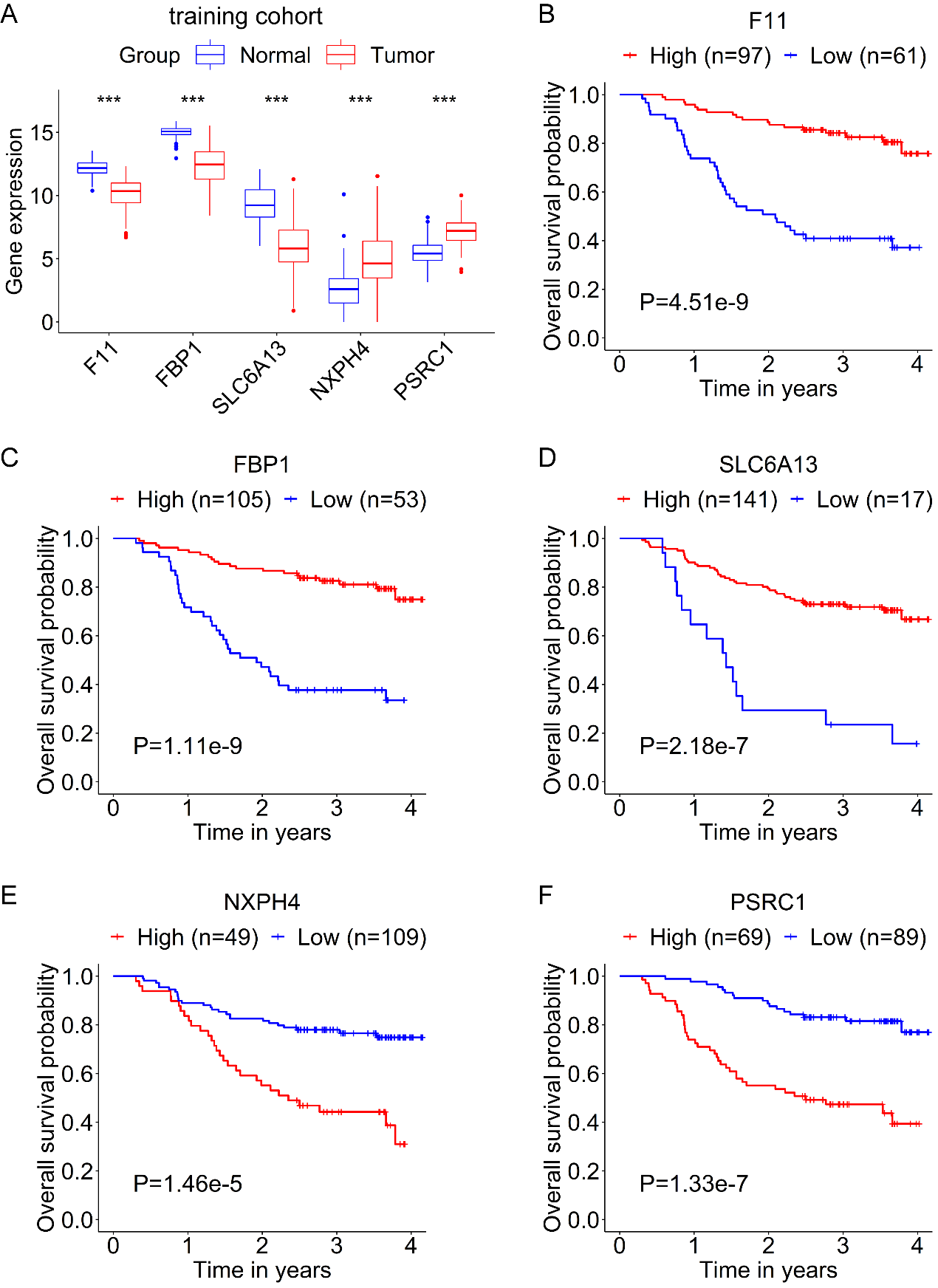


**Supplementary Figure 2. The differential expression and survival analyses of five RBP-related mRNAs in HBV-related HCC of the training cohort**

**(A)** The box plot shows the expression levels of five RBP-related mRNAs in the prognostic model between the normal and HBV-related HCC. Kaplan–Meier curves show the difference in OS between patients with the high and low expression of genes, including F11 **(B)**, FBP1 **(C)**, SLC6A13 **(D)**, NXPH4 **(E)**, and PSRC1 **(F)**.


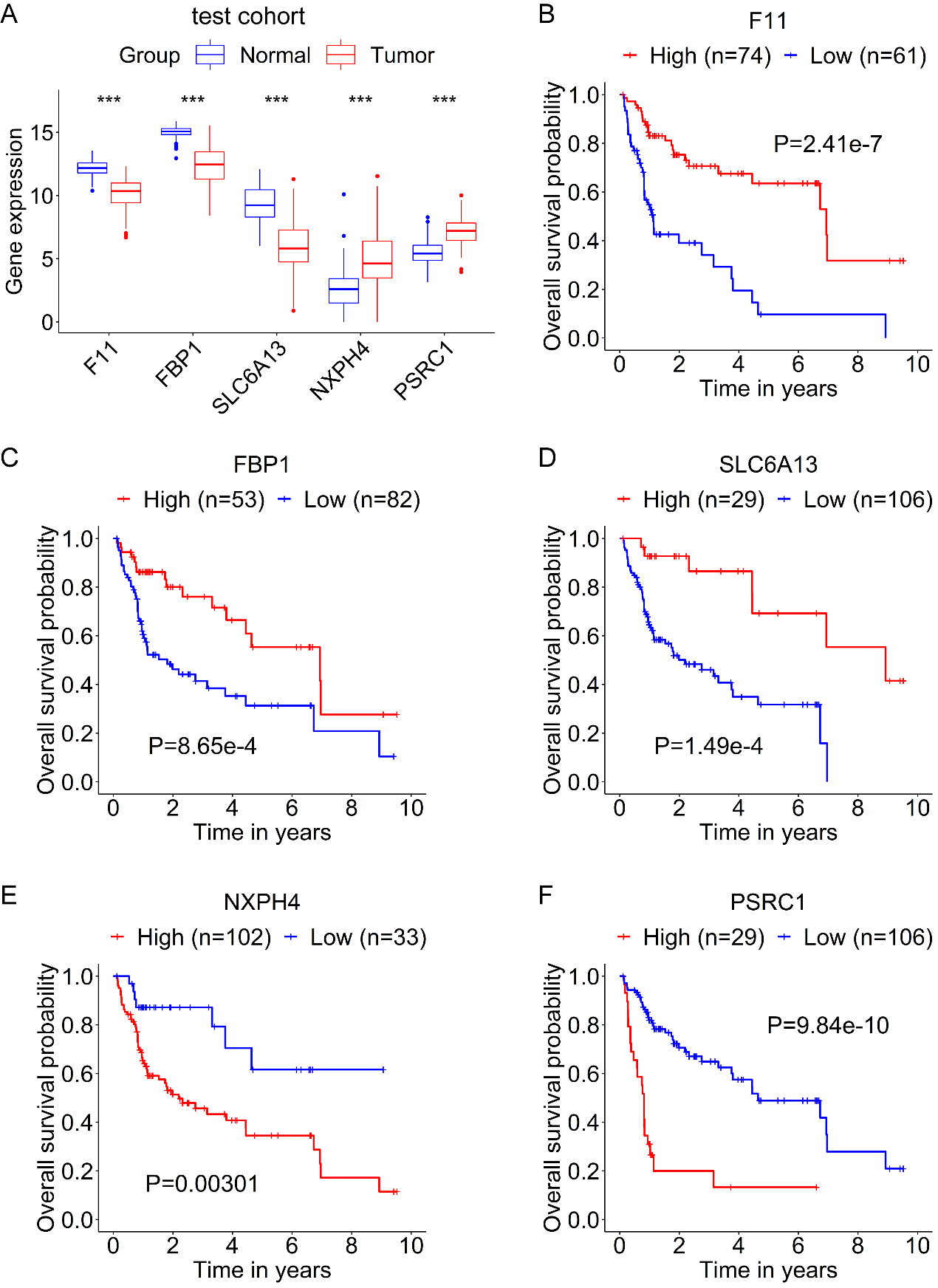


**Supplementary Figure 3. The differential expression and survival analyses of five RBP-related mRNAs in HBV-related HCC of the test cohort**

**(A)** The box plot shows the expression levels of 5 RBP-related mRNAs in the prognostic model between the normal and HBV-related HCC. Kaplan–Meier curves show the difference in OS between patients with the high and low expression of mRNAs, including F11 **(B)**, FBP1 **(C)**, SLC6A13 **(D)**, NXPH4 **(E)**, PSRC1 **(F)**.


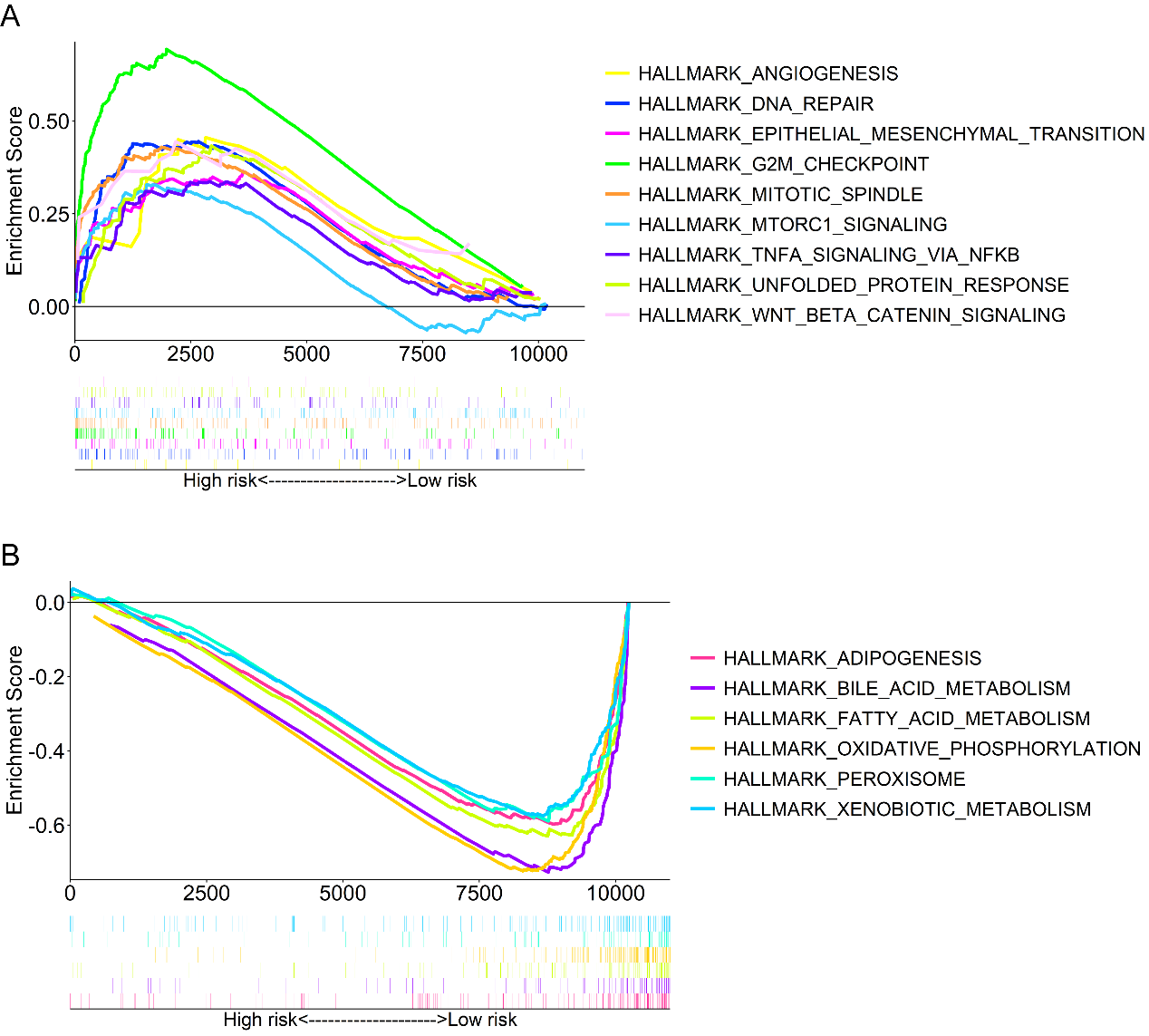


**Supplementary Figure 4. The GSEA analysis for proteins between the high- and low-risk groups in the training cohort**

**(A)** The hallmark pathways that were significantly enriched in the high-risk groups based on the proteomic data in the training cohort. **(B)** The hallmark pathways were significantly enriched in the low-risk groups based on the proteomic data in the training cohort.


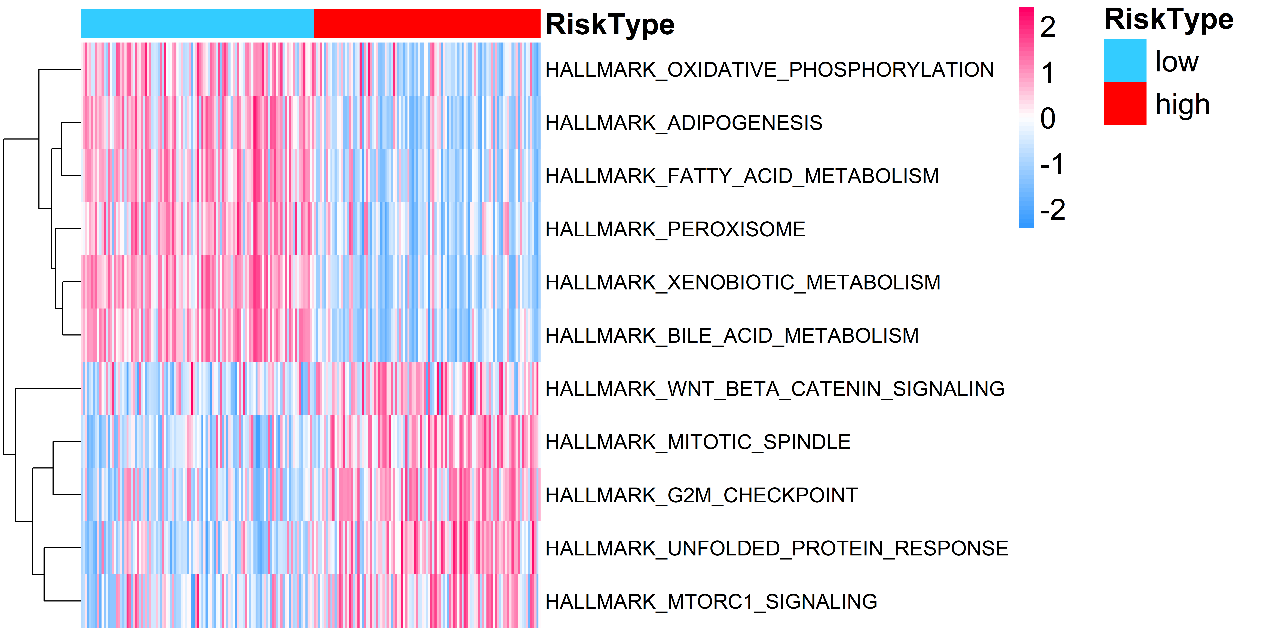


**Supplementary Figure 5. The GSVA for genes between the high- and low-risk groups in the validation cohort**

The hallmark pathways were significantly enriched in the high- and low-risk groups based on the gene expression data of the validation cohort.


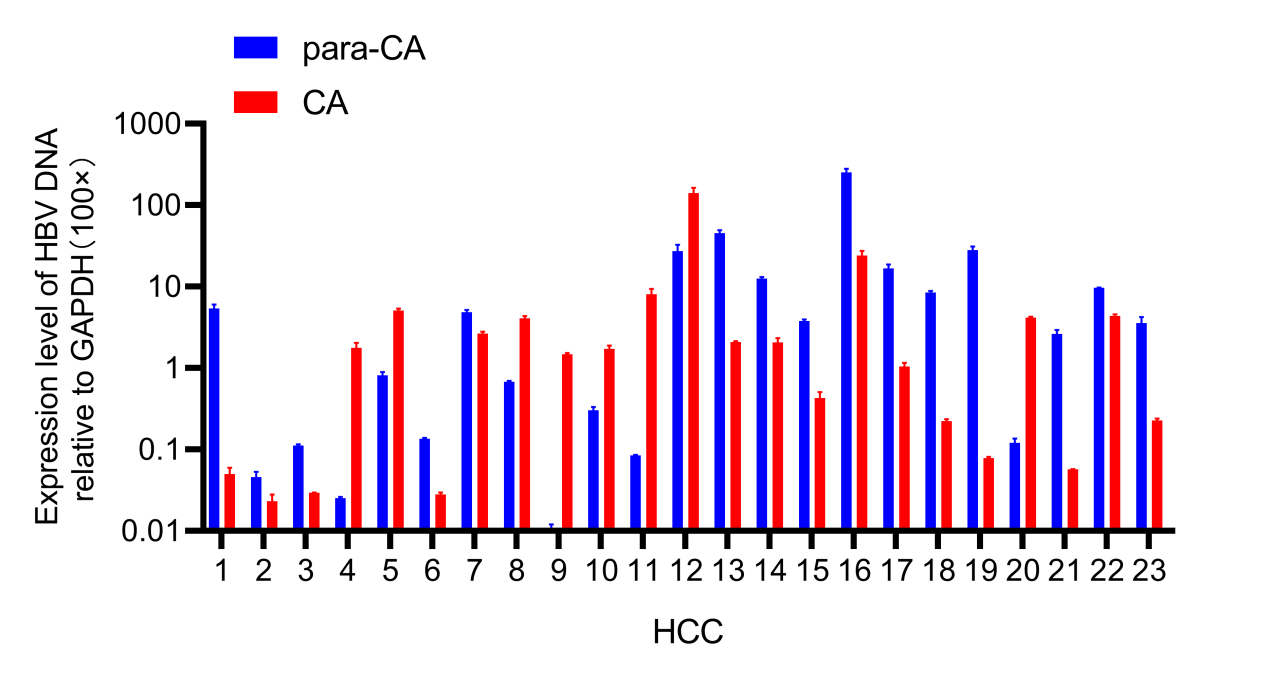


**Supplementary Figure 6. Hepatitis B virus (HBV) DNA detection in liver tissues of HBV-related HCC patients**

The total DNA was isolated from the liver tissues and HBV DNA was quantified by Real-time PCR. GAPDH was used as the normal control. The relative fold change of HBV DNA was calculated using the 2^-ΔCT^ method. CA: cancer tissues; para-CA: paracancerous tissues
